# Biological Evaluation of the Effect of Root Canal Sealers Using a Rat Model

**DOI:** 10.3390/pharmaceutics14102038

**Published:** 2022-09-24

**Authors:** Motoki Okamoto, Sayako Matsumoto, Kiichi Moriyama, Hailing Huang, Masakatsu Watanabe, Jiro Miura, Keita Sugiyama, Yujiro Hirose, Manabu Mizuhira, Nanako Kuriki, Julian G. Leprince, Yusuke Takahashi, Shigetada Kawabata, Mikako Hayashi

**Affiliations:** 1 Department of Restorative Dentistry and Endodontology, Osaka University Graduate School of Dentistry, 1-8 Yamadaoka, Suita, Osaka 565-0871, Japan; 2 Division for Interdisciplinary Dentistry, Osaka University Dental Hospital. 1-8 Yamadaoka, Suita, Osaka 565-0871, Japan; 3 Department of Oral and Molecular Microbiology, Osaka University Graduate School of Dentistry, 1-8 Yamadaoka, Suita, Osaka 565-0871, Japan; 4 Bruker Japan K.K. Nano Analytics Division, 3-9 Moriyacho, Yokohama, Kanagawa 221-0022, Japan; 5 DRIM Research Group & Advanced Drug Delivery and Biomaterials, Louvain Drug Research Institute, UCLouvain, 1200 Brussels, Belgium

**Keywords:** root canal sealer, biocompatibility, subcutaneous implantation, RNA-seq, micro-XRF

## Abstract

Gutta-percha points and root canal sealers have been used for decades in endodontics for root canal obturation. With techniques such as single cone methods, the amount of sealer is larger, making their properties more critical. However, relatively few reports have comprehensively evaluated their biological effects. To this end, we evaluated three types of sealers, zinc oxide-fatty acid-, bio-glass- and methacrylate resin-containing sealers were considered. Their biological effects were evaluated using a rat subcutaneous implantation model. Each sealer was loaded inside a Teflon tube and implanted subcutaneously in the backs of rats. Inflammatory cells were observed around all samples 7 days after implantation and reduced after 28 days. Our results revealed that all samples were in contact with the subcutaneous tissue surrounding the sealer. Additionally, Ca and P accumulation was observed in only the bio-glass-containing sealer. Furthermore, each of the three sealers exhibited unique immune and inflammatory modulatory effects. In particular, bio-glass and methacrylate resin sealers were found to induce variable gene expression in adjacent subcutaneous tissues related to angiogenesis, wound healing, muscle tissue, and surrounding subcutaneous tissue. These results may help to understand the biological impacts of root canal sealers on surrounding biological tissues, guiding future research and comparisons with new generations of materials.

## 1. Introduction

In endodontic treatments, various types of sealers for root canal obturation (RCO) are clinically applied along with gutta-percha (GP) points [1,2]. After root canal shaping and disinfection, RCO is performed to provide a hermetic seal to prevent re-infection. It has also been suggested that another role of RCO is to provide additional disinfection and reduce even further the amount of residual bacteria. Finally, the promotion of periapical healing has also been mentioned as a desirable action of RCO [3], for example by inducing the expression of anti-inflammatory mediators [4]. All these actions are supposed to increase the chance of treatment success over the long term, but without clinically demonstrated effects so far. For example, a recently published randomized clinical trial has failed to show superiority in terms of treatment success between an epoxy resin-based sealer and a calcium silicate one (17 months’ average follow-up) [5]. Overall, there is not much evidence supporting the use of one root canal sealer over another for RCO. A recent literature review reported no effect on apical periodontal tissue healing, but a large degree of bias was acknowledged [6]. Therefore, until more clinical data are made available, the lack of clinical evidence needs to be compensated by improving the knowledge and understanding of the biological effects of the most used commercially available sealers.

Cold lateral filling and warm vertical condensation methods using a combination of GP points and RCO sealers are frequently used filling strategies [7] and have been used for decades. Single cone techniques had disappeared and were considered obsolete, but gradually gained new popularity due to the ease of application combined with the marketing of new generations of sealers. With such techniques, the amount of sealer is larger, making their properties more critical. While numerous reports have evaluated the sealing properties of these materials, few reports have comprehensively evaluated their biological effects. It has been demonstrated that dental materials, such as RCO sealers, can affect specific gene expression, but these studies only focus on certain aspects of the materials evaluated. The present study is a comprehensive gene expression analysis of the effects of three types of subcutaneously implanted RCO sealers on the surrounding tissues of rats to reveal new aspects of the dental materials being evaluated.

Various types of RCO sealers have been developed over the last decades, the widely used being calcium-hydroxide-based and resin-based sealers, as well as zinc oxide sealers based on fatty acids [8]. Zinc oxide-fatty acids sealer has progressively replaced zinc oxide-eugenol sealer, which has high cytotoxicity. Additionally, resin-based sealers (epoxy- or methacrylate-based) have been introduced for their ability to adhere to root canal dentin, and for their increased radio-opacity [9]. Finally, newer generations of sealers labelled as “bio-active” have been developed and introduced, mainly tricalcium silicate sealers and sealers based on bio-glass, both having the potential to induce clinically favorable tissue responses [10,11]. Periapical tissue healing is a multi-factorial mechanism, involving bacteria removal on one hand, and promotion of inflammation resolution on the other hand; the use of bio-active sealers has been suggested as a way to promote healing, either though the intrinsic properties of the material or via their effect on dentin, such as growth factor release [12,13,14].

Root canal sealers come in contact with periapical tissues following the filling procedure. However, the biological effects of root canal sealers developed using various concepts and different main components are not completely clear. Specifically, a comprehensive analysis of the genes regulated by each root canal sealer has not been conducted. Therefore, the purpose of this study was to evaluate three types of commercially available sealers, i.e., zinc oxide-fatty acid-, methacrylate resin-, and bio-glass-containing sealers using a rat model. These materials were chosen since they are the most frequently used in the country where the project was conducted (Japan). The null hypothesis was that there is no difference in gene expression related to wound healing regulation and biocompatibility in tissues in which the three different root canal sealers evaluated were implanted.

## 2. Materials and Methods

### 2.1. Ethical Statement

All surgeries were performed under general anesthesia, and all efforts were taken to minimize pain or discomfort. This study was approved by the Ethical Guidelines Committee for Animal Care of Osaka University Graduate School of Dentistry (No. 29-028-0).

### 2.2. Root Canal Sealer Components and Material Preparation

**Figure 1:**
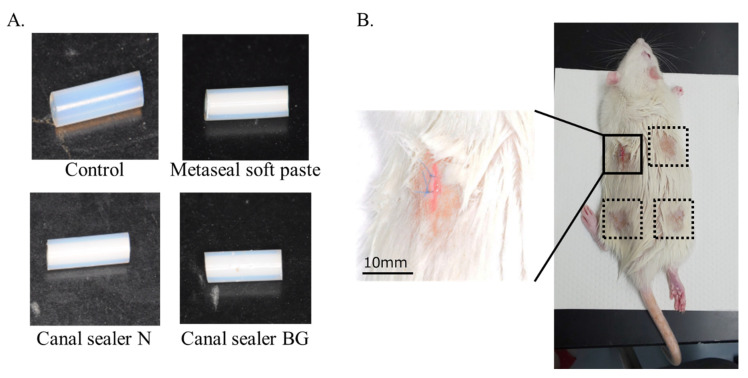
Subcutaneous implant model for root canal sealers. Three different kinds of materials were randomly implanted in four locations under the rat’s back skin. Representative image of each sample before implantation (**A**). Samples were randomly implanted into four locations subcutaneously (**B**). The magnified image shows sutures after implantation.

The following RCO sealers were obtained: Nishka Canal Sealer N^®^ as the zinc oxide-fatty acid sealer; Nishka Canal Sealer BG^®^ (Nippon Dental Chemical Co., Ltd.Yamaguchi, Japan) as the bio-glass-containing sealer; Metaseal soft paste^®^ (Sun Medical Co., Ltd. Shiga, Japan) as the methacrylate resin sealer. The components of the RCO sealer used in this study are presented in Table 1. A cylindrical polytetrafluoroethylene (PTFE) tube (Flon Industry, Tokyo, Japan) with an inner diameter of 1 mm was sterilized with ethylene oxide gas and prepared to a length of ~3.5 mm. Each mixed RCO sealer was then placed in a cylindrical PTFE tube (Figure 1A). These cylinders were over-filled with sealer, and the excess was scraped off to ensure a similar volume of material, and a comparable surface of contact with the subcutaneous tissue. 

### 2.3. Subcutaneous Implantation Procedure

Fourteen 9-week-old Wistar rats weighing 200–230 g (CLEA Japan Inc., Tokyo, Japan) were used for the in vivo experiments. Before material implantation, anesthesia was induced with 0.3 mg/kg medetomidine hydrochloride (Domitol; Meiji Seika Pharma Co., Ltd., Tokyo, Japan), 4.0 mg/kg midazolam (Dormicum; Astellas Pharma Inc., Tokyo, Japan), and 5.0 mg/kg butorphanol (Vetorphale; Meiji Seika Pharma Co., Ltd., Tokyo, Japan). Body hair was then partially removed from the back of the rat where material implantation was planned. Careful dissection was performed and a 10 mm incision line was established (Figure 1B), parallel to the epidermal and muscular layers, to retain the cylinders within the subcutaneous connective tissue. There was little or no bleeding. Prepared root canal sealers were randomly implanted into four locations subcutaneously. Empty tubes without the root canal sealer were used as controls. Tubes from the three prepared RCO sealers were implanted in subcutaneous tissue at a depth of ~2 mm and sutured with two threads of 6-0 polypropylene thread (Prolene, Ethicon, New Brunswick, NJ, USA). Stitches were removed after 7 days and the animals were kept for the duration of each evaluation. The animals were monitored for any abnormal weight loss, feeding, water deprivation, or self-injurious behavior; in any of these events, the experiment would be terminated.

### 2.4. Histological Evaluation of the Tissue around the Root Canal Sealer

The subcutaneously implanted cylinders were retrieved after 7 and 28 days. The collected specimens were fixed in 4% paraformaldehyde at 4 °C for 24 h and then embedded in paraffin. After paraffin embedding, the tubes were removed, serial sections of longitudinal cross-sections (6 µm thick) were prepared, and hematoxylin and eosin (HE) staining was applied.

### 2.5. Microstructure Analysis Using Scanning Electron Microscopy (SEM)

The RCO sealer-filled cylinders were subcutaneously implanted, and after 14 days, the entire subcutaneous tissue around the specimen was harvested. Paraformaldehyde (2%) and glutaraldehyde (2.5%) were used for fixation. Postfixation was performed in 2% osmium tetroxide. Tissue specimens were dehydrated in ascending ethanol series and embedded in epoxy resin (Quetol-812, Nissin EM Tokyo, Japan). The surfaces of each specimen were polished well with diamond abrasive sheets (30–0.3 μm grain size, Maruto Instruments, Tokyo, Japan). Backscattered electron images were acquired using SEM (TM-3000, Hitachi High-Tech, Tokyo, Japan).

### 2.6. Elemental Analysis of the Subcutaneous Tissue Affected by the Root Canal Sealer

Rats with each root canal material implanted subcutaneously were kept for 14 days, and elemental analysis of the neighboring subcutaneous tissue affected by the RCO sealer was performed via micro-X-ray fluorescence spectroscopy (micro-XRF), based on previous reports [15]. Implanted tubes containing each material were collected, chemically fixed with 2% paraformaldehyde and 2.5% glutaraldehyde, and then dehydrated in a graded ethanol series. The samples were then embedded in epoxy resin (Quetol-812, Nissin EM, Tokyo, Japan). The resin samples were then split longitudinally, and the surfaces were polished and observed under a micro-XRF spectrometer (M4 TORNADO, Bruker, Berlin, Germany). Based on the data obtained, elemental maps were created for calcium (Ca), phosphorus (P), strontium (Sr), aluminum (Al), silicon (Si), and bismuth (Bi). The basic parameters for the micro-XRF were as follows: voltage 50 kV; current 600 µA; pixel size 4 µm; exposure time 12 min under dry conditions. The elemental mapping was performed using Esprit software (Bruker, Berlin, Germany), and the distribution of these elements according to intensity was displayed.

### 2.7. RNA Extraction, RNA Sequencing (RNA-seq) and Data Analysis

Comprehensive gene expression analysis of biological tissues via RNA-seq was performed 10 days after subcutaneous implantation of the RCO sealers, based on previous reports [16]. The animals were anesthetized and the subcutaneous tissue around the implanted tube was excised. Immediately after harvesting, the tissue was immersed in RNA preservation solution (RNA later, Sigma-Aldrich, St. Louis, MO, USA)) and divided into small portions using a homogenizer (Power Masher Ⅱ, BioMasher® SP, nippi, Tokyo, Japan) on ice. Total RNA was extracted using the RNeasy Mini RNA isolation kit (Qiagen, N.V, Hilden, Germany) for RNA-seq analysis. RNA integrity was assessed with a 2100 bioanalyzer (Agilent Technologies, Santa Clara, CA, USA). Directional RNA-seq libraries were created using the TruSeq stranded mRNA sample Prep Kit (Illumina Inc., San Diego, CA, USA), following the manufacturer’s recommendations. Library sequencing was carried out using the Illumina NovaSeq 6000 system with 100-base-pair (bp) single-end reads. Raw reads were deposited into the DNA Data Bank of Japan (DDBJ) sequence read archive (DRA, accession number: DRA011981). Data were generated in the standard Sanger FastQ format and Phred-type quality scores Q30 were used for quality trimming. RNA-seq reads were mapped against a rat reference genome (rnor6) using the commercially available CLC Genomics workbench (CLC Bio, Aarhus, Denmark). Differential expression and global analyses of RNA-seq expression data were performed using iDEP (PMID: 30567491), with the RPKM value of each sample determined. EdgeR log transformation was used for clustering and PCA (iDEP). Hierarchical clustering was visualized using the average linkage method with correlation distance (iDEP). The overlaps of differentially expressed genes (DEGs) were extracted using Venn diagrams. We classified the DEGs into GO terms using DAVID Bioinformatics Resources (PMID: 17576678). Transcriptomic (RNA-seq) data are summarized in Supplemental Data Sets 1, 2, and 3.

### 2.8. Gene Ontology Analysis

To classify the DEGs into Gene Ontology (GO) terms, we uploaded DEGs to the online DAVID Bioinformatics Resources website (PMID: 17576678). The results of the gene ontology analysis were visualized with the number of genes and adjusted *p*-values. 

## 3. Results

### 3.1. Histopathological Evaluation of the Tissue around the Root Canal Sealer

**Figure 2:**
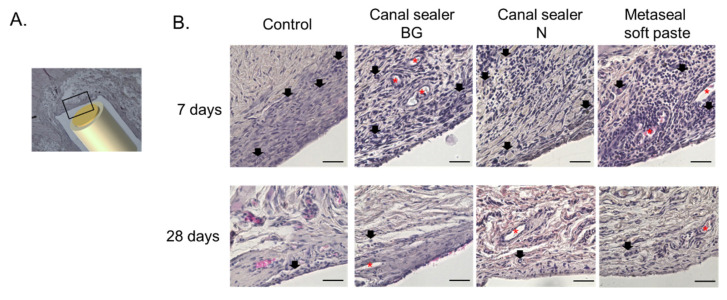
Histological evaluation of the subcutaneous tissue around each root canal sealer. The subcutaneous tissue around the RCO sealer was observed histologically (n = 6 per material). The observation site was the area that was in contact with the RCO sealer (**A**). Representative images of each material 7 and 28 days after subcutaneous implantation are shown (**B**). Black arrows and red asterisks indicate inflammatory cells and vascular structures, respectively. RCO: root canal obturation.

To histologically evaluate the effect of the RCO sealer on the subcutaneous tissues, they were removed 7 and 28 days after implantation (Figure 2A). As a characteristic of all RCO sealers, a large number of inflammatory cells were observed in the tissue sections within the initial 7 days after implantation, and the inflammatory cells disappeared after 28 days (Figure 2B). For the BG and Metaseal soft paste groups, luminal structures that appeared to be vessels lined by endothelial cells were observed as early as 7 days after transplantation.

### 3.2. Microstructure Analysis Using SEM

**Figure 3:**
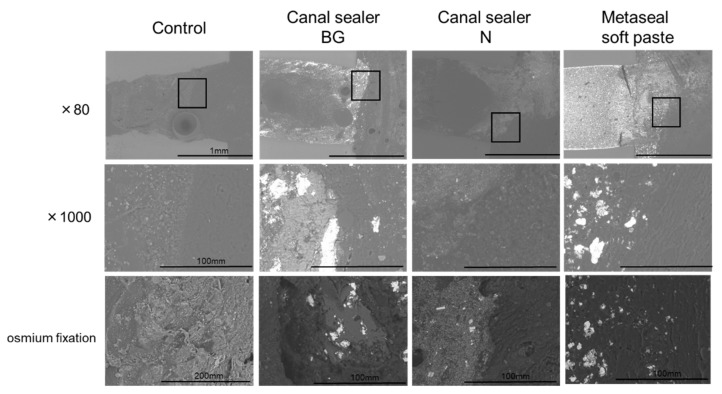
Scanning electron microscopy. The subcutaneous tissue around the RCO sealer was resin-embedded and analyzed (n = 3 per material). The image with high magnification is the enlargement of the black box in the image with low magnification. The bottom images correspond to osmium fixation, which was used to enable subcutaneous tissue observation. RCO: root canal obturation.

Subcutaneously implanted RCO sealer-filled cylinders and the surrounding tissue were collected after 14 days for microstructural observation via SEM (Figure 3). Soft tissue observation revealed that all RCO sealers had slightly dissolved, disintegrated, or incorporated into the subcutaneous tissue in the contact area. No voids were observed between the materials and the subcutaneous tissues, which indicated no rejection.

### 3.3. Elemental Analysis of the Subcutaneous Tissue Affected by the Root Canal Sealer

**Figure 4:**
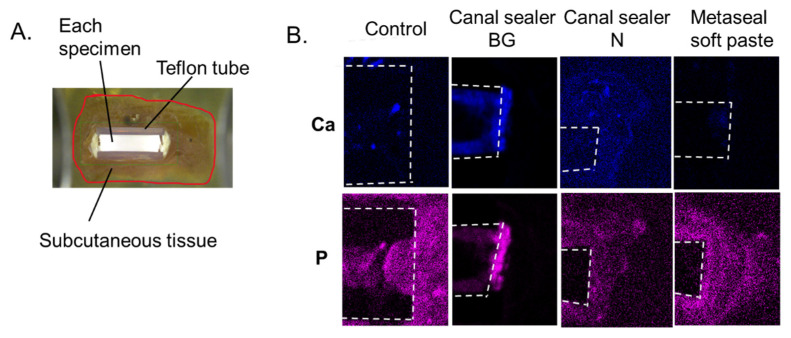
Micro-XRF analysis. The subcutaneous tissue around the RCO sealer was resin-embedded and analyzed using micro-XRF (**A**), (n = 3 per material). Red lines indicate the outline of the subcutaneous tissue. Elemental mapping of Ca and P obtained via micro-XRF is shown (**B**). The white dotted line shows the outline of the Teflon tube filled with the root canal filler. XRF: X-ray fluorescence spectroscopy. RCO: root canal obturation.

A representative image of the Metaseal soft paste group is shown in Figure 4A. Micro-XRF analysis showed the characteristic elemental distribution in each sample (Figure 4B). As a result of elemental analysis mapping, a specific detection of P and Ca in the material surface was observed only in the BG group. Specific detection of other elements such as Fe, Zn, S, Si, Al, O, Sr, Bi, F, C, Cl, and Mg were not observed. The detection of Ca and P on the surface of the BG sealer is a specific finding because this mapping method applied shading based on the distribution of elements detected in the area of imaging.

In other groups, the distribution of elements was comparable to that detected as background in the subcutaneous tissue and was not observed. 

### 3.4. Transcriptome and Gene Ontology Enrichment Analysis of Differentially Expressed Genes in the Subcutaneous Tissues

**Figure 5:**
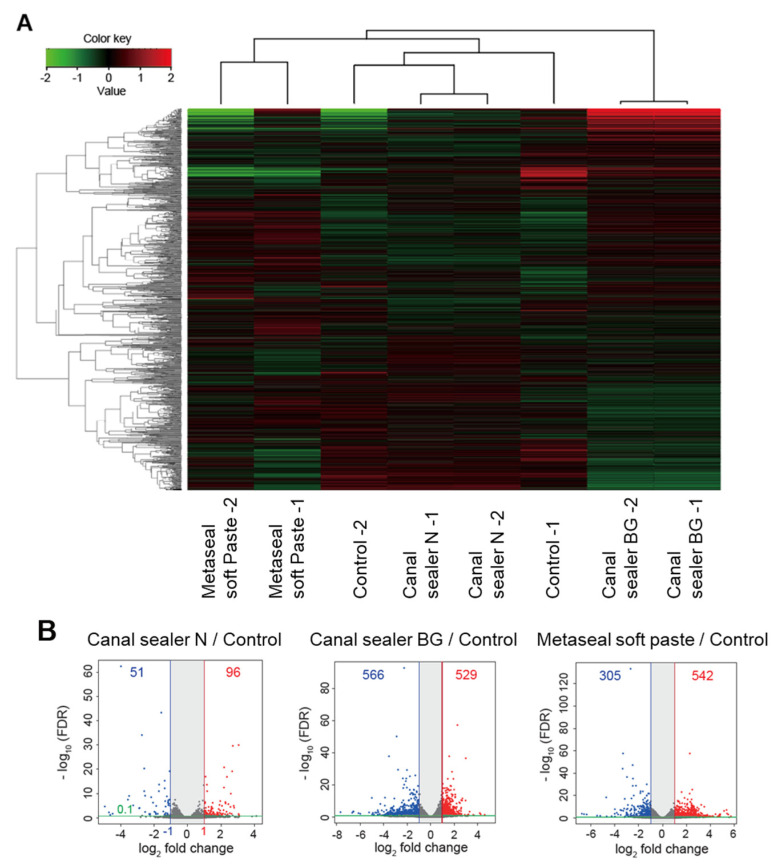
RNA-seq results. Heat map shows the clustering of top 10,000 genes that contribute to the differences in each group (**A**). Each column represents a material and each row a gene. Clustering was performed using iDEP with edgeR log transformation of read counts. Hierarchical clustering was illustrated using the average linkage method with correlation distance. Color-coding was based on edgeR log-transformed read counts. Color key indicates Z-scores, which are displayed as relative values for all tiles within all samples. Green indicates the lowest level, black an intermediate level, and red the highest level of expression. Volcano plots present the differential expression of genes between the Canal Sealer N, Canal Sealer BG, or Metaseal soft paste groups and the control group (**B**). The plots show differences in gene expression under the conditions indicated in each figure. Colored circles indicate significantly upregulated (red) and downregulated (blue) genes (absolute log2 fold change > 1; adjusted *p* < 0.1).

A heatmap showing the clustering of the top 10,000 genes contributing to the differences in each RCO sealer group showed that the Canal Sealer N group showed a gene expression pattern relatively similar to the control. In contrast, the Metaseal soft paste and the Canal Sealer BG groups showed different gene expression patterns (Figure 5A). These results were compared to controls (empty tubes) using a Volcano plot. This confirmed that Canal Sealer N showed relatively similar behavior to the controls, while the Metaseal soft paste and Canal Sealer BG had a greater effect on gene expression in the subcutaneous tissue compared to controls (Figure 5B). Briefly, Canal Sealer N promoted the expression of 96 genes and suppressed 51 compared to controls, while Canal Sealer BG and Metaseal soft paste promoted the expression of 529 and 542 genes, respectively, and suppressed 566 and 305, respectively, compared to the control.

**Figure 6:**
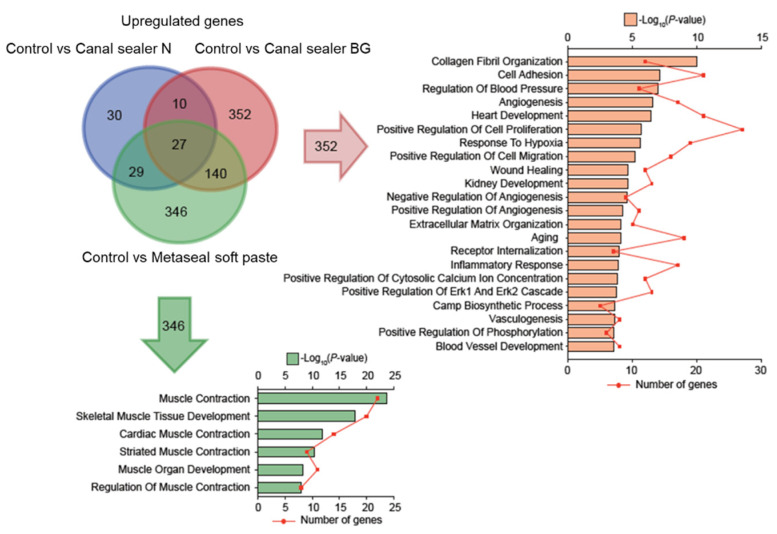
Unique GO terms of differentially upregulated genes in the Canal Sealer BG or Metaseal soft paste groups. The three-way Venn diagram illustrates the overlapping upregulated genes among groups. The top 22 unique GO terms in the Canal Sealer BG group are listed in red. The top six unique GO terms in the Metaseal soft paste group are listed in green.

**Figure 7:**
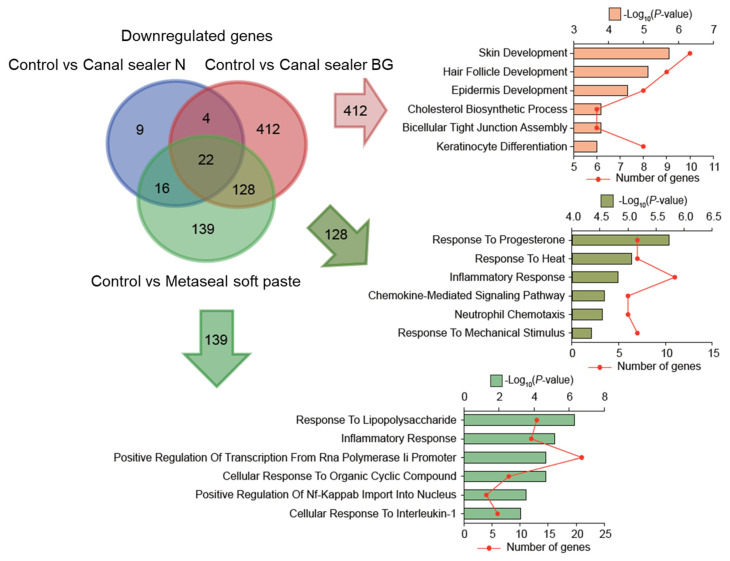
Unique GO terms of differentially downregulated genes in the Canal Sealer BG and Metaseal soft paste groups.

Venn diagrams were drawn to represent genes with increased (Figure 6) or decreased (Figure 7) expression in each group. Their function based on GO classification was determined, according to the differentially expressed genes between different types of sealers and the control. Since the gene expression pattern for Canal Sealer N was relatively similar to the control, only the comparison of the two other materials to the control were presented in the form of a Venn diagram. 

Regarding upregulated genes (Figure 6), the Canal Sealer BG group showed notably increased expression of genes related to cell adhesion, angiogenesis, wound healing, cell migration, cell migration, and inflammatory response, whereas the Metaseal soft paste group showed increased expression of genes related to muscle tissue contraction and development.

As regards the function of genes whose expression was suppressed, the downregulation of genes related to inflammatory responses was common to both materials (Canal Sealer BG and Metaseal soft paste) compared to control. The Metaseal soft paste group also showed downregulation of the genes related to the suppression of inflammation, which was different from the Canal Sealer BG group. A three-way Venn diagram illustrates overlapping of downregulated genes between groups. The top six unique GO terms are listed in each section.

Transcriptome (RNA-seq) data are reported in detail in Supplemental Data Sets 1, 2, and 3.

## 4. Discussion

Zinc oxide-eugenol sealers were developed in the 1930s and have long played a central role in endodontic RCO [17,18]. However, the cytotoxicity of eugenol, which was incorporated as an instrumental chelating material, has attracted attention, and RCO sealers using various fatty acids as chelating materials have been developed and widely used [19]. In the present study, Canal Sealer N was used as a zinc oxide-fatty acid sealer.

In addition, many RCO sealers, such as salicylic acid-based, silicone-based, glass ionomer-based, tricalcium silicate-based (MTA/bioceramic), methacrylate resin-based, and epoxy resin-based sealers have been developed and are widely used worldwide, with each dental company making improvements that can be applied in endodontic treatment [8]. In the present study, Metaseal soft paste was used as a methacrylate resin sealer. Another RCO sealer evaluated in this study was the bio-glass-containing sealer BG. Although it contains fatty acids, it is considered one of the bio-ceramic sealers, and clinical studies using this RCO sealer have reported that it has high biocompatibility [20]. These sealers were chosen because they are frequently used in the country where the project was conducted (Japan).

There have been many in vitro and in vivo reports evaluating many root canal filler sealers, focusing on their sealing and antibacterial properties, which can cause endodontic failures, as well as their cytotoxicity, which is related to their biocompatibility [21,22,23,24]. In addition to the above roles, RCO sealers are expected to have a positive effect on the tissue at an early stage, such as by promoting wound healing of apical tissue. There have been reports examining the biological effects of RCO sealers containing bio-ceramics [25,26]. There have been reports on the biological effects of RCO sealers focusing on specific genes and signal pathways [27].

In endodontics, RCO is an important procedure that intends to maintain sterile conditions in root canals in the long term and thereby maintain the results achieved by the disinfection and root-canal shaping procedure. RCO sealers are considered biomaterials since they interact with biological tissues for a medical purpose. These materials are sometimes referred to as “bioactive,” although this terminology is actually a misuse of the term in all commercially available materials today [28]. However, these materials can have profound effects on the biological tissues in contact or in close proximity with them, as was demonstrated in the present work, particularly for the resin- and bio-glass-based materials.

Paradoxically, relatively few studies extensively investigating the biological effects of RCO sealers are available. In particular, there were no reports including a comprehensive genetic analysis. In this study, we evaluated the biological effects of three types of root canal filler sealers (zinc oxide-fatty acid, bio-glass, and methacrylate resin), for which little information was available.

Histological evaluation via HE staining showed an inflammatory reaction in all samples 7 days after treatment (Figure 2). However, the inflammatory reaction was reduced in all groups 28 days after implantation, and no additional inflammatory reaction was observed compared to the control. The reduction in inflammatory cells was a result of a similar phenomenon reported in a rat subcutaneous model that evaluated different types of RCO sealers than those used in the present study [29]. These results suggest that the RCO sealers used in the present study have high biocompatibility. This was also supported by SEM images (Figure 3). After the specimens were implanted under the dorsal skin and the surrounding subcutaneous tissue was removed and epoxy-resin embedded, the microstructure was observed via SEM. The images showed that the subcutaneous tissue was in contact with the RCO sealer, and there were no images showing detachment from the root canal sealer (Figure 3). Although we could not find a report on SEM observation of subcutaneous tissue in contact with the RCO sealer after subcutaneous implantation, the results reinforce the high biocompatibility of the evaluated sealers, as their biocompatibility could be observed without being affected by the histopathology section preparation process, and no voids were observed at the sealer interface. Regarding angiogenesis/luminal structures involved in wound healing, which comprise one of the bioactive effects, luminal structures surrounded by squamous epithelial cells were observed in the Canal Sealer BG and Metaseal soft paste groups 7 days post operation (Figure 2). An important role of calcium silicate/tricalcium silicate in endodontics is biomineralization, which is the deposition of hydroxyapatite at the contact surface of the material and tissue [30]. Micro-XRF results showed that Ca and P accumulation was only observed in the Canal Sealer BG group (Figure 4), which is considered to be hydroxyapatite induced by contact with bio-glass and subcutaneous tissue. This hydroxyapatite observed in micro-XRF is considered to have strong biological effects [30]. There are limited reports evaluating RCO sealers with XRF [31], and the target of evaluation is the composition of the sealer itself, not the precipitate resulting from the reaction with the simulated body fluid. This is the first report to visually capture the reaction products of bio-glass-containing sealer and subcutaneous tissue using XRF. 

Angiogenesis was also observed in the subcutaneous implantation model. These results were supported by RNA-seq results, which confirmed that Canal Sealer BG predominantly increased the expression of genes related to wound healing and angiogenesis (Figure 6). Metaseal soft paste, on the other hand, increased the expression of genes related to muscle tissue formation and differentiation (Figure 6). These effects are thought to be related to muscle tissue adjacent to the subcutaneous tissue, and the fact that this material was found to promote wound healing in tissues adjacent to the subcutaneous tissue was considered a positive effect in that it did not inhibit healing in the local environment. However, further investigation is needed to determine what components and mechanisms are responsible for this effect, and most of all if such effect would be verified in a periapical environment.

The results of this comprehensive genetic analysis showed that the control and Canal Sealer N had a limited effect on gene expression in the subcutaneous tissue compared to the other sealers. A heatmap showing the clustering of the top 10,000 genes contributing to the differences between each RCO sealer group showed that the Canal Sealer N group showed a gene expression pattern that was similar to that of the control, while Metaseal soft paste and Canal Sealer BG showed different gene expression patterns between groups. Although the clustering was material-specific, the technical error of the gene analysis in this study was considered to be small.

Additionally, the results of RNA-seq showed that several genes were predominantly upregulated (Figure 6) and several were predominantly downregulated in the Canal Sealer BG group (Figure 6), especially related to the inflammatory response. Genes associated with suppressed inflammatory response or immune response were those common to the Canal Sealer BG and Metaseal soft paste groups and those more specifically suppressed by resin-based sealers (Figure 7). These genes may be involved in the regulation of the inflammatory response, which is characteristic of Canal Sealer BG and Metaseal soft paste. The expression of genes related to inflammatory response and immune response was also downregulated in the Canal Sealer N group, which showed a fluctuation in the expression of a small number of genes, suggesting that these sealers may have a unique inflammation-modulating effect (Supplementary Figure S1). 

The methacrylate resin sealer used this study, Metaseal soft paste, is a highly biocompatible sealer [32], and there was no excessive residual inflammatory reaction, suggesting that the effect of the residual monomer was small. This sealer is said to initiate polymerization at the wet dentin interface with the hydrophilic monomer and a hydrophilic amino acid-based polymerization initiator and heal in the presence of moisture. In subcutaneously operated tissues, the cylindrically loaded RCO material expanded after healing (Figure 4A). Resin-based sealers generally shrink with polymerization, but Metaseal soft paste showed water absorption expansion with fluid or a moist environment. This phenomenon may contribute to the containment of bacterial invasion in clinical practice, but a detailed study is needed to determine the extent to which this phenomenon occurs in an environment with less fluid and blood flow than the subcutaneous tissue.

In the present study, three available RCO sealers developed based on their respective concepts were evaluated in detail. This is the first report to examine the comprehensive gene expression profile in tissues affected by RCO sealers. The results provide not only basic data for collecting the biological effects of currently available RCO sealers, but also for the development of next-generation RCO sealers with bioactive effects. RNA-seq metadata is publicly available on DDBJ and can be used by researchers worldwide. Although a systematic review indicated that the available evidence does not indicate that the material used for RCO has any effect on periapical healing [6], it is clear that materials have different effects on peri-material tissues at the genetic level. This is therefore useful in pre-clinical material evaluation and development. In the future, we will also investigate root canal filler sealers, such as calcium silicate sealers, which are being increasingly used, and contribute to the accumulation of data that will lead to a better understanding of the available materials and to the development of next-generation root canal filler sealers.

As a limitation of this study, a subcutaneous implantation model was used to investigate the biological effects of RCO sealers [33], which does not reproduce the clinical environment in which the sealers will be used. Recently, spatial genetic analysis using histopathology sections has become possible [34]. Therefore, adapting this method to root canals in animal experiments and performing a comprehensive analysis for each of the periapical root tissues would provide additional important data.

## 5. Conclusions

The zinc oxide-fatty acid, bio-glass-containing and methacrylate resin sealer used in this study all have high biocompatibility without inducing excessive inflammatory responses. The bio-glass and methacrylate resin sealer groups were found to have variable gene expression in adjacent subcutaneous tissues related to angiogenesis, wound healing, and muscle tissue, the tissue surrounding subcutaneous tissue. In contrast, the eugenol-based sealer group showed a relatively similar gene expression pattern to the control. The present work may help to understand the biological effects of root canal sealers on surrounding biological tissues, which will guide their future developments and help compare them with new generations of materials. 

## Figures and Tables

**Table 1:** Material proportions.

Materials		Main Composition
**Canal sealer N** zinc oxide-fatty acid sealer	Fatty Acid based Zinc Oxide Sealer (Nippon Shika Yakuhin Co., Ltd.)	olive oil, zinc oxide, bismuth hypocarbonate, magnesium oxide, fatty acid, rosin, ester gum. Lot number J7S
**Metaseal soft paste** methacrylate resin sealer	Resin-based root canal sealer (MetaSEAL Soft Paste, SUN MEDICAL)	methacrylic esters (HEMA, 4-META, others), water, photopolymerization initiator, contrast agent, organic filler. Lot number VRA
**Canal sealer BG** Bio-glass contained sealer	Fatty Acid based Zinc Oxide Sealer containing Bioactive Glass (Nippon Shika Yakuhin Co., Ltd.)	magnesium oxide, purified water, calcium silicate glass, silicon dioxide, fatty acids, bismuth hypocarbonate. Lot number K3K

## Data Availability

The data presented in this study are available on request from the corresponding author. Raw reads of RNA-seq were deposited into the DNA Data Bank of Japan (DDBJ) sequence read archive. Publicly available datasets were analyzed in this study. This data can be found here: (DRA, accession number: DRA011981).

## Supplementary Materials

The following supporting information can be downloaded at: https://www.mdpi.com/article/10.3390/pharmaceutics14102038/s1, Supplementary Figure S1: GO terms associated with differentially up- or downregulated genes in response to Canal Sealer N. Supplementary Dateset1, Supplementary Dateset2, Supplementary Dateset3.
