# Supplementary material for: Biological Evaluation of the Effect of Root Canal Sealers Using a Rat Model
Source: Pharmaceutics. 2022 Sep 24;14(10):2038. doi: 10.3390/pharmaceutics14102038 (PMC9606985; doi:10.3390/pharmaceutics14102038)
Supplement: Supplementary file 1 [file pharmaceutics-14-02038-s001.zip › pharmaceutics-1917447-Supplementary.pdf]

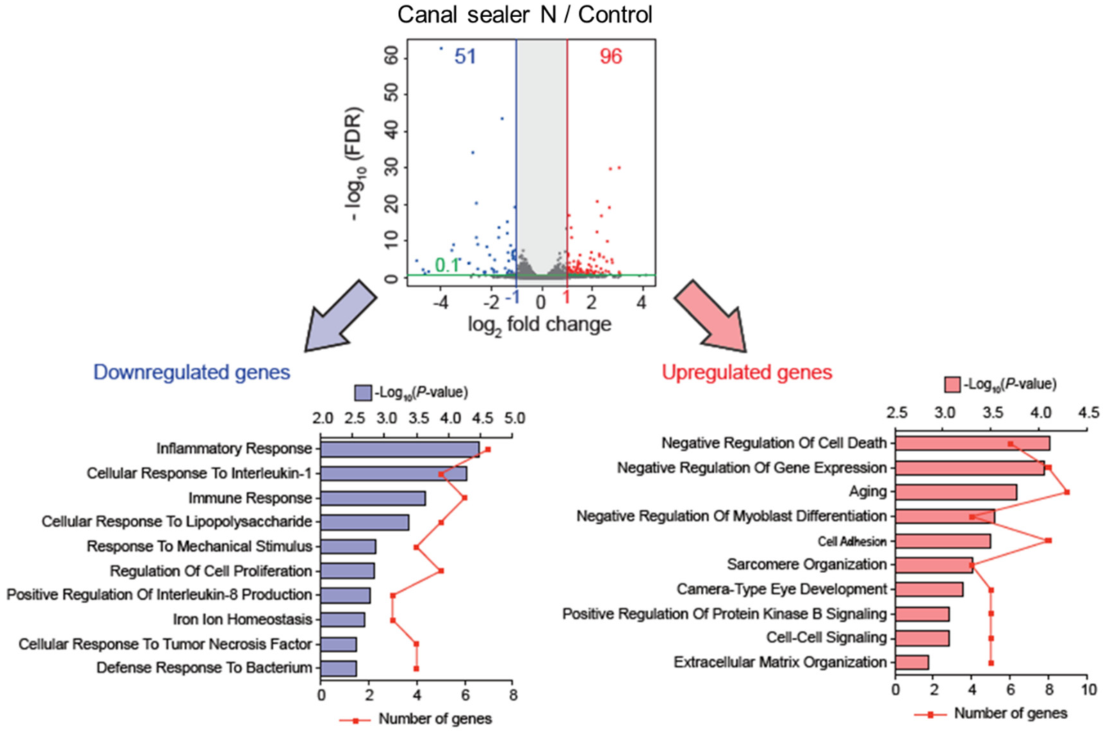

Supplemental Figure S1. GO terms of differentially upregulated or downregulated genes in Canal sealer N.

Volcano plots show gene expression differences under the comparison with control group. Colored circles indicate significantly upregulated (red) and downregulated (blue) genes (absolute log2 fold change, >1; adjusted $p < 0.1$). Top 6 unique GO terms are listed in each section. The expression of genes related to inflammatory response and immune response was also downregulated in the Canal Sealer N group, which showed a fluctuation in the expression of a small number of genes, suggesting that these sealers may have a unique inflammation-modulating effect.
